# Capabilities of Modern Semiconductor Gamma Cameras in Radionuclide Diagnosis of Coronary Artery Disease

**DOI:** 10.3390/diagnostics11112130

**Published:** 2021-11-17

**Authors:** Michał Błaszczyk, Zbigniew Adamczewski, Anna Płachcińska

**Affiliations:** 1 Department of Quality Control and Radiological Protection, Medical University of Łódź, Czechosłowacka 8/10 Street, 92-216 Łódź, Poland; mich.blaszczyk00@gmail.com (M.B.); anna.plachcinska@umed.lodz.pl (A.P.); 2 Department of Nuclear Medicine, Medical University of Łódź, Czechosłowacka 8/10 Street, 92-216 Łódź, Poland

**Keywords:** coronary artery disease, CZT camera, dynamic SPECT, myocardial flow reserve

## Abstract

This paper presents a review of the literature concerning the clinical application of modern semiconductor (CZT) gamma cameras in the radioinuclide diagnosis of coronary artery disease. It contains information on the diagnostic efficacy of myocardial perfusion studies performed with those cameras compared with the widely used scintillation (Anger) cameras, an overview of their effectiveness in comparison with coronary angiography (also fractional flow reserve) and currently available clinical results of a myocardial flow reserve measured with a dynamic SPECT study. Introduction of this imaging modality to the measurement of a myocardial flow reserve aims to facilitate access to this type of study compared to the less available and more expensive PET method used so far.

## 1. Introduction

CZT semiconductor gamma cameras form a new generation of imaging devices in nuclear medicine. In standard Anger cameras, which have been used for decades in nuclear medicine to image a distribution of radiopharmaceuticals in the patient body, detectors are equipped with sodium-iodide crystals [NaI (Tl)]. They transform gamma radiation into electrical impulses, but this phenomenon is indirect. The interaction of gamma rays with the crystal structure of NaI (Tl) forms light photons (scintillations), which are then converted into a beam of electrons that are amplified by the photomultiplier system. Such detection systems are characterized by large dimensions and sub-optimal resolution.

On the other hand, in semiconductor detectors introduced several years ago to radionuclide diagnostic imaging, a direct conversion of gamma rays into an electrical impulse is applied, providing information about the location as well as the energy of interaction in the detector. These detectors, defined by the symbol CZT (cadmium-zinc-telluride), outperform the NaI (Tl) scintillation crystal in terms of their stopping power of gamma rays, and are also characterized by higher energy and spatial resolution. The replacement of scintillation crystals with semiconductor detectors allowed for the introduction of cameras dedicated to cardiology, with better imaging parameters than devices equipped with scintillation crystals [1].

These instruments are equipped with many small detectors in the camera head, that can image the heart simultaneously from different perspectives—no rotation around a patient is required. This property allows the shortening of study acquisition time or, alternatively, to reduce the activity of the administered radiopharmaceutical [2,3,4], thus reducing the exposure of a patient to ionizing radiation.

**Figure 1:**
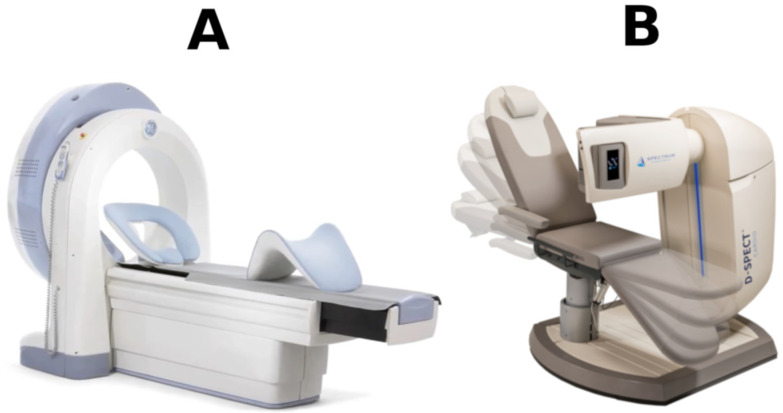
Cardiac—centered CZT SPECT cameras: (**A**) Discovery NM 530c by General Electric and (**B**) D-SPECT by Spectrum Dynamics.

At present, two dedicated CZT cardiac cameras, SPECT, are available (Figure 1).

Discovery NM 530c by General Electric and D-SPECT by Spectrum Dynamics. They are both equipped with a crescent-shaped head adjusted to the patient’s chest. They differ in the arrangement of detectors (5 mm thick and with a pixel size of 2.5 mm × 2.5 mm) and the way of collimation (multi pinhole vs. parallel hole).

During study acquisition with the Discovery NM 530c camera, a patient is placed in the supine or prone position. The camera head contains 19 stationary CZT detectors that can be placed close to the heart, and acquire gamma rays simultaneously at different angles. Pinhole collimators with the area of interest located on the left ventricular myocardium are used.

D-SPECT camera allows the study of patients in a sitting or supine position. A camera head contains nine columns of detectors which, rotating at some angle, sweep the studied area. Each column consists of four CZT detectors. The angle at which each of them is located at the time of interaction with the gamma ray is taken into account during a tomographic reconstruction procedure [5,6].

Due to no need to rotate the detection system around a patient during SPECT study acquisition, this examination can be performed in a dynamic way, which was not possible with the classic Anger (A-SPECT) cameras.

The aim of this study is to present the current capabilities of semiconductor CZT gamma cameras in cardiological diagnostics.

## 2. Myocardial Perfusion Study

Myocardial perfusion study has a well-established position in the non-invasive diagnosis of coronary artery disease [7]. According to the guidelines of the European Society of Cardiology (ESC), in patients with an uncertain diagnosis of this disease, myocardial perfusion study is one of the recommended diagnostic methods [8]. Moreover, this study has a prognostic value and helps in choosing the optimal therapeutic method.

### 2.1. CZT-SPECT vs. A-SPECT

A high agreement of results was demonstrated between perfusion images obtained with the CZT-SPECT and A-SPECT cameras. In a multicenter study involving 168 patients, Esteves et al. [9] (Table 1) compared images obtained with the Discovery NM 530c and standard gamma cameras. The images made it possible to detect or exclude ischemia with high, 91.9% agreement (compared to 92.5% agreement, obtained when the same studies acquired with a standard camera were assessed twice, *p* = 0.99). Sharir et al. [10] demonstrated, by means of a multicenter study involving 238 patients, high concordance of quantitative assessments of total perfusion deficits (for D-SPECT and A-SPECT in the stress and rest studies linear correlation coefficients equaled 0.95 and 0.97, respectively) with good agreement in three vascular territories (kappa coefficients for LAD, LCX and RCA were 0.73, 0.73 and 0.70, respectively, the concordance in detecting defects in each of the three vascular areas exceeded 90%).

In addition, Verger et al. [11] verified, on a group of 276 patients, the agreement of study results obtained with a semiconductor and standard gamma cameras in studies applying three different protocols: using a standard and low activity of [99mTc] Tc-MIBI, as well as [201Tl] thallium. In all cases, a high agreement was obtained between results on both types of cameras; with standard activity [99mTc] Tc-MIBI—98%; low—86% and using [201Tl] thallium—92%. There was also a high agreement (over 85%) between the A-SPECT and CZT-SPECT cameras in terms of the percentages of abnormal study results, ischemia and permanent perfusion defects. Other authors of studies on smaller groups of patients also demonstrated high agreement between results of studies on both discussed types of cameras [11,12,17,28,29].

However, the consistency of the results was not so high in all patient groups. Mannarino et al. [12] examined a group of women with low and intermediate probability of coronary artery disease using both types of cameras. There was no significant correlation between summed stress scores (SSS)—r = 0.18, *p* = 0.06, and a significant but weak (48%) correlation between total perfusion deficit (TPD) obtained as a result of semi-automatic comparison of individual images with normal databases. The discrepancy concerned the perfusion of anterior wall. In A-SPECT, weaker uptake of the radiotracer was detected more often in this area, which was not observed in CZT-SPECT. Based on the normal wall thickening in the gated study, all results suspected of ischemia were classified as normal. CZT-SPECT was considered a more useful technique in this group of patients due to its greater effectiveness in excluding perfusion abnormalities.

### 2.2. Diagnostic Efficacy of CZT Cameras

In a meta-analysis of 40 studies including a total of 7734 patient studies on the diagnostic efficacy of CZT-SPECT and A-SPECT, Cantoni et al. [13] showed, in relation to coronary angiography, that CZT-SPECT fulfills its role properly in the diagnosis of coronary artery disease. Its diagnostic efficacy was slightly higher than that of A-SPECT (sensitivity and specificity compared to coronary angiography were 89% and 69% versus 85% and 66%, respectively, while the areas under the ROC curve amounted to 0.89 and 0.83). The accuracy of studies using CZT-SPECT was slightly, although statistically significantly, higher than that of studies with A-SPECT (*p* < 0.05). This is explained by better spatial resolution and the resulting higher contrast of images obtained with semiconductor gamma cameras. Authors of previous meta-analyzes involving studies on semiconductor cameras combined with coronary angiography (Nudi et al. [14]—2092 patients and Zhang et al. [15]—2350 patients) obtained similar results regarding the sensitivity and specificity of studies in the detection of coronary artery disease, without showing significant differences between the two CZT cameras used in the research. Moreover, Gimelli et al. [16] showed, on the material of 695 patients, that a CZT camera allows for the effective detection of CAD in patients with a multivessel disease. Areas under the ROC curves obtained on the basis of semi-quantitative measures—summed stress scores (SSS) in patients with two- and three-vessel disease, 0.83 and 0.79, respectively, did not differ significantly from the area under the ROC curve for single-vessel patients—0.80. This method allowed for the proper detection of the extent of coronary artery disease in 64% of patients. In addition, regional perfusion defects corresponding to the location of the three major coronary arteries were detected highly efficiently (areas under the ROC curves for LAD, LCX, and RCA amounted to 0.90, 0.88, and 0.87, respectively). The article of Gimelli et al. indicates an advantage of semiconductor over Anger cameras in terms of the effective detection of patients with a multivessel disease. There are many previous reports informing about difficulties in diagnosing such patients due to the relative nature of a radiopharmaceutical distribution in images, and also in the proper determination of the extent of the coronary artery disease using conventional cameras [30,31].

As shown by the two previously mentioned meta-analyzes of the results of CZT-SPECT perfusion studies [14,15], the specificity of those studies in detecting coronary artery disease is lower than their sensitivity (69% and 72% vs. 84% and 84%, respectively). The relatively high rate of false-positive results is attributed, as is the case with conventional cameras, to the attenuation of radiation in the patient body, which is largely related to obesity [17]. In order to improve the quality of studies, attempts are made to correct the attenuation. Another approach to solve this problem is placing a patient in different positions during study acquisition, assuming that the filling of perfusion defects due to a change in the patient position indicates their false nature.

Attenuation correction (AC) aims to eliminate artifacts resulting from radiation absorption in a patient body. On the basis of studies using a hybrid semiconductor camera integrated with a CT (Discovery NM/CT 570c), van Dijk et al. [29] showed that AC increases diagnostic confidence (the percentage of correct stress images increased from 45% of uncorrected—NC, up to 72% of attenuation corrected—AC, and up to 67% while considering both sets—corrected and uncorrected). A subsequent follow-up did not reveal a higher incidence of major cardiac events in patients whose NC studies were considered more normal than those with AC. The authors conclude that the use of AC allows for more frequent giving up of the rest study, and thus limiting the exposure to ionizing radiation to the dose of about 1.2mSv.

However, only a few sites in the world have a hybrid cardiac CZT camera. Attempts are being made to obtain the same information using a CT not integrated with a gamma camera. The question of whether AC is necessary for a reliable assessment of myocardial perfusion imaged by the CZT camera has not yet been resolved.

Caobelli et al. [18] report that, in comparison with results of coronary angiography, external CT attenuation correction increases the specificity of the CZT-SPECT study (specificity for the AC and NC studies were, respectively: in the assessment of a whole myocardium perfusion—100% vs. 40%; in the assessment of vascular perfusion—LAD: 63% vs. 36%, LCX: 70% vs. 33%; RCA: 81% vs. 19%). AC significantly increased the diagnostic accuracy of the global myocardial score (*p* = 0.01) as well as in the RCA vascular territory (*p* = 0.02), but not in the LAD (*p* = 0.35) and LCX areas (*p* = 0.08).

On the other hand, Esteves et al. [19] claim that the attenuation correction of CZT-SPECT images does not significantly affect their visual interpretation. Moreover, CT exposes patients to an additional radiation dose and, due to a possible patient motion resulting in a SPECT and CT misalignment, may affect the credibility of the examination.

As already mentioned, another method of dealing with artifacts resulting from the absorption of radiation in the patient body is to perform the examination in two different positions. Mirshavalad et al. [20], in a meta-analysis of studies using standard and semiconductor cameras, showed on two separate groups of patients that the prone position study—1490 patients—allows for similar sensitivity (83% vs. 86%) and higher specificity (79% vs. 67%) than the study in the supine position—1138 patients. The pooled sensitivity and specificity of the prone position in detecting the right coronary artery territory defects were 70% and 84%. Nishiyama et al. [21] showed, on a group of 276 patients, that taking into account images from both positions (supine + prone) significantly improves the perfusion interpretation in terms of specificity (*p* = 0.02) and accuracy (*p* = 0.04) in relation to the supine position: sensitivity, specificity and accuracy: 85% vs. 78%, 85% vs. 50%, 85% vs. 76%, respectively.

Study acquisition in prone and supine positions is possible only with the Discovery NM 530c camera. In the case of D-SPECT, it is possible to acquire studies in the upright, supine and bikerlike positions. Combined assessment of images acquired in the sitting and supine position (upright + supine), in comparison with the results of coronary angiography, provided a sensitivity and specificity of 94% and 86%, respectively, compared to studies acquired only in the upright (91% and 59%) or only supine (88% and 73%) positions; areas under the ROC curves differed statistically significantly (0.94 vs. 0.88 and 0.89, *p* < 0.05) [22]. The forward leaning bikerlike position decreased a distance between the heart and the detector and eliminated attenuation artifacts in relation to the sitting and supine position [32].

The Discovery NM 530c camera is prone to artifacts in very obese patients resulting from a small field of view and a pinhole collimation geometry [33,34].

In D-SPECT cameras this problem does not occur and, in doubtful situations, caused by diaphragmatic artifacts, it is possible to perform a study in sitting and supine positions [12,22].

It is worth emphasizing that the CZT camera, due to its higher sensitivity, provides greater flexibility in the selection of a study protocol. Compared to classic cameras, one can shorten the acquisition time of a study with preserved radiopharmaceutical activity, reduce activity or apply both, to a lesser extent. By administering a lower activity patient radiation dose can be reduced [9]. As reported by Einstein et al. [35], it is even possible to reduce the effective dose to 1mSv in the case of normal stress studies when the other parts are not necessary.

Among the limitations of CZT cardiac cameras, the difficulties in observing patient movement during study acquisition are stressed [36]. This movement can have a significantly negative impact on reconstructed images. Software for Anger cameras makes it possible to observe patient movement, for example by displaying raw images in a cinematic mode or by observing the discontinuities of heart coordinates. Patient movement can be easily corrected before a reconstruction procedure.

However, in the case of CZT cardiac cameras, due to their different geometry, correction of the patient movement, although possible, is much more difficult and, above all, time-consuming. This limits its application to cases significantly suspected of patient movement during study acquisition.

A high cost of those devices is also stressed, especially because they are dedicated to a strictly defined type of studies [36].

## 3. Left Ventricular Contractility

### 3.1. Global Contractility

Gated acquisition of a myocardial perfusion SPECT study with a patient ECG signal (GSPECT) increases the diagnostic value of the study, providing the assessment of global and regional left ventricular (LV) contractility [37].

Studies have been conducted to compare CZT-GSPECT in terms of left ventricular functional parameters (end-systolic volume—ESV, end-diastolic volume—EDV, and ejection fraction—EF) with gold standard magnetic resonance imaging (MRI) and with A-GSPECT. The authors of three reports on this subject—Cochet et al. [38], Giorgetti et al. [39] and Claudin et al. [40]—obtained high or fairly high values of the correlation between results of EDV measurements (0.71; 0.90; 0.94, respectively) and ESV (0.88; 0.94; 0.96, respectively) compared to MRI. However, these volumes were significantly underestimated (e.g., in the work of Giorgetti et al. the underestimation was 39.5 mL for EDV, and 15.4 mL for ESV), as a result of the lower resolution of CZT-SPECT cameras than MRI. Nevertheless, these differences did not significantly affect global ejection fraction (EF) assessment due to its relative value. In general, high correlations were demonstrated between the values of the ejection fraction obtained from CZT-GSPECT and MRI (0.81, 0.84 and 0.88, respectively).

It should be emphasized that, due to the higher resolution of CZT cameras, the results of the left ventricular volume measurements are less underestimated than with conventional cameras.

There was also a paper (Sala et al. [41]) comparing left ventricular volume and ejection fraction measurements using the CZT (Discovery) camera and magnetic resonance imaging. In this work, [201Tl] thallium was used as the radionuclide, and a Corridor 4DM software (INVIA) was used for volume measurements. Although values of both the volume and the ejection fraction were highly correlated with the results from magnetic resonance (EDV, ESV and EF 0.91, 0.95 and 0.86, respectively), the EF was systematically significantly underestimated [48 (15%) vs. 55 (18%); *p* < 0.001], and ESV was systematically overestimated [84 (71) vs. 72 (63) mL; *p* = 0.001]. Differences between these results and what was observed in all other studies are difficult to interpret reliably. The differences are likely to be attributed to different software used to process the study, different (lower) radiation energy of [201Tl] thallium vs. [99mTc] technetium, and possibly also the study reconstruction parameters.

### 3.2. Regional Contractility

Cochet et al., comparing the usefulness of the CZT camera with MRI in terms of regional (in 17 segments) left ventricular contractility, using wall motion and wall thickening, showed moderate agreement of both imaging methods. The correlation between myocardium regional contractility measures was 0.49 (*p* < 0.0001). Although a systematic difference between CZT and MRI measures was small (−1.4 mm), the 95% limits of agreement were quite wide (−6.9 mm to 4 mm). Even less encouraging results were obtained for the myocardium thickening: a correlation of 0.48 (*p* < 0.0001), a systematic difference of −41%, and limits of agreement between 108% and 26% [38].

Despite these results, Coupez et al. [42] found the usefulness of the segmental assessment of left ventricular myocardium thickening in the detection of post-infarction scar, thanks to the good correlation between severe impairment of thickening detected with the CZT camera and delayed post-contrast segment enhancement in MRI. On the other hand, Claudin et al. [40], comparing left ventricular contractility divided into 17 segments and applying a visual assessment on a 3-point scale: akinesia or dyskinesia, hypokinesia and normal contractility, obtained high agreement (kappa coefficient—0.72, strict agreement—87%) between the gated perfusion study on the CZT camera and the MRI.

## 4. Myocardial Flow Reserve

As mentioned earlier, the SPECT myocardial perfusion study is a widely used diagnostic method. Despite its well-established position in the diagnosis of coronary artery disease, it has some limitations due to the relative nature of perfusion images. In patients with multivessel disease, there is a risk of visualizing ischemia only to the extent of a vessel with the greatest narrowing. In the case of three-vessel disease, where blood flow is evenly reduced throughout the myocardium, a perfusion image may sometimes seem normal.

In such cases, a more effective diagnosis is provided by positron emission tomography (PET). In addition to the relative image of perfusion, this study allows us to obtain fully quantitative indices of blood flow through the myocardium. Dynamic imaging of an intravenously administered radiopharmaceutical in a bolus form, flowing through the right heart, lungs and left heart and then accumulating in the left ventricular muscle, allows the determination of the myocardial blood flow in mL/(min × g) under stress conditions and at rest. Based on these values, a myocardial flow reserve (MFR) is calculated [43]. It is most often defined as a ratio of the blood flow in myocardium under hyperemic conditions to the blood flow at rest. Positron emission tomography, using water with radioactive oxygen [^15^O]O-H_2_O, is considered the gold standard in the assessment of myocardial blood flow. This tracer is characterized by free diffusion with an extraction coefficient independent of the flow rate and thanks to this, it most reliably reflects myocardial blood flow. PET coronary flow reserve takes into account changes of myocardial perfusion resulting from both ischemia at epicardial coronary vessels and microcirculation level [23,44,45,46]. Coronary flow reserve assessment can also be performed using [^13^N] N-ammonia; however, as in the case of [^15^O]O-H_2_O, a significant limitation in using this tracer in everyday clinical practice is the need to have an on-site cyclotron.

In recent years, [^82^Rb]rubidium, an analog of potassium, has been used for this purpose. It is of generator origin, so using it does not require having a cyclotron.

A normal value of MFR is considered to be at least 2. It has been shown that a significant reduction below 1.5 is associated with a higher probability of future coronary events [47]. Only slightly reduced values may be a consequence of small vessel disease, which is not necessarily accompanied by ischemia of epicardial arteries [47]. Moreover, Ziadi et al. [48] demonstrated that MFR is an independent predictor of three-vessel coronary disease and significantly complements the classical study of myocardial perfusion.

Despite the reliable assessment of coronary blood flow in PET studies using the aforementioned radiopharmaceuticals, the costs and availability are still factors that prevent their widespread use.

As mentioned earlier, the recently introduced technological solutions for the construction of gamma cameras (CZT-SPECT) allow for dynamic acquisition of the study, as well as obtaining quantitative flow indices similar to those provided by dynamic PET study. Several reports show that results indicating the clinical usefulness of these studies have already been obtained. In the WATERDAY study, Agostini et al. [23] compared the results of myocardial flow reserve (MFR) values obtained with CZT-SPECT using [^99m^Tc]Tc-tetrofosmin and PET with radioactive water [^15^O]O-H_2_O, in comparison with the fractional flow reserve—FFR obtained during coronary angiography. MFR obtained with both nuclear methods had similar values both for the entire myocardium and for each of the vascular areas (MFR: total—2.84 vs. 2.64; LAD—2.67 vs. 2.52; LCX—2.8 vs. 2.7; RCA—2.77 vs. 2.99). High indices of diagnostic efficacy were obtained (sensitivity, specificity and accuracy of 83.3%, 95.8%, 93.3%, respectively) in identifying patients with reduced MFR measured in PET and 58.3%, 84.6%, and 81.1%, respectively, for detecting hemodynamically significant stenosis (FFR ≤ 0 8).

Bouallegue et al. [26], performing a study of coronary flow and reserve in 23 patients with previously diagnosed multivessel coronary disease, using [^99m^Tc]Tc-tetrofosmin and comparing the results with coronary angiography showed a strong (r = 0.70) and statistically significant (*p* < 0.001) linear correlation between the number of critically narrowed vessels and the value of the global reserve. Moreover, the values of the reserve were statistically significantly lower in patients with three-vessel disease compared to patients without such extensive disease [1.57 (0.41) vs. 2.17 (0.50), *p* = 0.01], while no such relationship was found for TPD (total perfusion deficit), nor for the global ejection fraction. The authors also showed that regional values of the reserve differed statistically significantly between critically stenosed and non-stenosed vessels [1.72 ± 0.5 vs. 2.51 ± 0.66, *p* < 0.001].

Shiraishi et al. [49] presented an interesting combination of results of a standard myocardial perfusion study with the myocardial flow reserve using the CZT-SPECT camera in the detection of coronary artery disease. In a study using [^201^Tl]thallium, they compared MFR measurements with the angiographic image of 125 patients with normal SPECT perfusion (SSS ≤ 3) who were referred for coronary angiography for reasons other than perfusion defects in this study. Of these, 40 (32%) had one vessel disease (critical 1 vessel stenosis), 24 (19.2%)—two vessel disease, and 14 (11.2%)—three vessel disease. The MFR index in patients with significant stenosis in the coronary arteries was significantly lower than in patients without such changes [2.85 ± 0.73 vs. 2.47 ± 0.53, *p* < 0.001]. The area under the ROC curve for the MFR used to detect patients with coronary artery disease was 0.75 (*p* < 0.001), while the sensitivity and specificity for the cut-off value of 2.7 were 77% and 66%, respectively. The authors concluded that a myocardial flow reserve study may be helpful in the diagnosis of latent, balanced coronary artery disease not visible in standard perfusion images.

The usefulness of MFR in the diagnosis of coronary artery disease has also been demonstrated in other studies relating to coronary angiography (both morphological and functional—FFR). Zavadovsky et al. [24] showed a significant correlation between MFR and FFR (r = 0.66, *p* = 0.01), as well as sensitivity and specificity for the MFR cut-off value of 1.48 equal to 69.2% and 93.3%, respectively. De Souza et al. [25], comparing the myocardial perfusion and reserve study with the results of coronary angiography showed that patients with abnormal myocardial perfusion were characterized by reduced MFR values [2.01 (1.48–2.77) vs. 2.94 (2.38–3.64), *p* = 0.002]. Mean MFR values were significantly lower in patients with a percentage deficit of perfusion > 10% than in patients with a deficit < 5% (*p* = 0.02). Lower regional MFR indices were also observed in significantly stenosed vessels than in unchanged vessels [1.81 (1.19–2.67) vs. 2.75 (2.13–3.42), *p* < 0.001]. A moderate but significant correlation was also observed between the regional MFR values and the maximum degree of stenosis in the corresponding coronary artery (r = −0.348, *p* < 0.001). Miyagawa et al. [27], in a study of 153 patients, demonstrated a significantly lower global MFR in three-vessel patients [1.18 (1.01–1.35)] than in single-vessel patients [1.46 (1.16–1.76), *p* = 0.003], as well as a higher area under the curve ROC for global MFR (0.812, *p* < 0.001) than for SSS (0.757, *p* = 0.002), LVEF (0.699, *p* = 0.03), TPD (0.699, *p* = 0.06) and SDS (0.646, *p* = 0.3). The correlation of regional MFR values with the FFR was significant, albeit moderate (r = 0.62, *p* = 0.008). Most importantly, they showed that the global MFR cut-off of 1.3 showed high diagnostic indices, 93.3% sensitivity and 75.9% specificity in detecting three-vessel disease.

The cut-off values of the MFR presented in the above review, significantly different from each other, result from the use of different blood flow models (one-compartment vs. net retention model) and mainly from the use (or not) of corrections for lower extraction rates in the myocardium of technetium-99m labeled cardiological radiopharmaceuticals (MIBI, tetrofosmin) compared with radiopharmaceuticals used in the PET studies (empirical Renkin–Crone correction).

## 5. Conclusions

In conclusion, it should be emphasized that the myocardial perfusion study performed with CZT camera, despite certain limitations, strengthens its position as an effective diagnostic method. Comparable or even higher diagnostic values, the possibility of shortening the examination time or reducing the activity of the radiopharmaceutical (and thus reducing the patient’s radiation exposure) are important factors in favor of introducing these devices into everyday practice. The innovative construction of these devices, allowing for a dynamic SPECT study, is an extremely promising factor, which will probably make a myocardial flow reserve study more available, due to the widespread use and lower costs of SPECT studies compared to PET, used so far only for this purpose. SPECT MFR studies are especially promising for the proper diagnosis of patients with multivessel disease.

## Figures and Tables

**Table 1:** The most important information along with a list of literature on the clinical use of CZT cameras in the diagnosis of coronary artery disease.

Esteves et al. [9]	Perfusion	A-SPECT ^1^ vs. CZT-SPECT ^2^	92% Agreement in Detection of Ischemia
Sharir et al. [10]			High concordance of TPD ^3^, concordance in vascular territories >90%
Verger et al. [11]			High >85% concordance for MIBI ^4^ and Thallium
Mannarino et al. [12]			Women with low to intermediate prob. of CAD ^5^—no correlation between SSS ^6^ values
Cantoni et al. [13]		CZT-SPECT vs. coronary angiography	Diagnostic efficacy of CZT-SPECT slightly higher than of A-SPECT (AUROC ^7^ 89% vs. 83%)
Nudi et al. [14]			Detection of CAD: sensitivity. 0.84, specificity 0.69
Zhang et al. [15]			Detection of CAD: sensitivity 0.84, specificity 0.72
Gimelli et al. [16]			Effective detection of patients with mutivessel CAD
Van Dijk et al. [17]		AC ^8^	Increase in specificity (0.45 to 0.67)
Caobelli et al. [18]			increase in specificity (0.40 to 0.100)
Esteves et al. [19]			AC does not affect visual interpretation
Mirshavalad et al. [20]		Two patient positions	Higher specificity in prone than in supine position (0.86 vs. 0.67)
Nishiyama et al. [21]			Supine + prone—increase in specificity vs. supine only (0.85 vs. 0.50)
Nakazato et al. [22]			Upright + supine vs. upright (sensitivity 0.94 vs. 0.86, specificity 0.91 vs. 0.59)
Agostini et al. [23]	MFR ^9^	Comparison with PET ^10^	Agreement in entire myocardium and in each of vascular territories
Agostini et al. [23]		Comparison with angiography or FFR ^11^	High specificity (0.85) and accuracy (0.81) in detection of haemodynamically significant stenosis (FFR ≤ 0.8).
Zavadovsky et al. [24]			correlation between MFR and FFR (r = 0.66, *p* = 0.01)
De Souza et al. [25]			Lower regional MFR indices in significantly stenosed than in unchanged vessels [1.81 vs. 2.75, *p* < 0.001].
Bouallegue et al. [26]		3-vessel disease	MFR lower in patients with 3-vessel disease than without it: 1.57 vs. 2.17, *p* = 0.01
Myagawa et al. [27]			Lower global MFR in 3- than in 1-vessel patients [1.18 vs. 1.46 *p* = 0.003], high diagnostic indices, 0.93 sensitivity and 0. 76 specificity in detecting 3-vessel disease

^1^ A-SPECT—SPECT (single photon emission computed tomography) study performed with Anger gamma camera; ^2^ CZT-SPECT—SPECT study performed with semiconductor cadmium zinc telluride gamma camera; ^3^ TPD—Total perfusion deficit; ^4^ MIBI—methoxy-isobutyl-isonitrile, technetium-99m labeled cardiological radiopharmaceutical; ^5^ CAD—coronary artery disease; ^6^ SSS—summed stress score; ^7^ AUROC—area under receiver operating characteristic curve; ^8^ AC—attenuation correction; ^9^ MFR—myocardial flow reserve; ^10^ PET—positron emission tomography; ^11^ FFR—fractional flow reserve.
